# The Brainard District Medical Society

**Published:** 1879-06

**Authors:** J. A. Walker

**Affiliations:** Secretary


### ARTICLE IX.

### THE BRAINARD DISTRICT MEDICAL SOCIETY.

The Brainard District Medical Society met April 24. Present: Drs. J. W. Newcomer, H. B. Brown, M. Hurst, S. T. Hurst, J. D. Whitley, W. S. Watson, Chas. B. Maclay, A. I. Maclay, P. L. Dieffenbacher, W. P. Walker, I. N. Ellsberry, J. P. Walker, O. P. Crane, J. A. Walker, J. W. Spear and A. M. Bird.

The following new members were elected: Drs. Chas. B. Maclay and A. I. Maclay, of Delavan; Dr. J. W. Downey, of Topeka; and Dr. W. P. Walker, of Walker's Grove.

Officers elected for the ensuing year as follows: President, Dr. J. W. Newcomer; Vice Presidents, Drs. J. D. Whitley, I. N. Ellsberry and H. B. Brown; Secretary, Dr. J. A. Walker; Treasurer, Dr. A. M. Bird.

The retiring President, Dr. J. P. Walker, read his valedictory address, on the advancement made by the profession within the quartery century—after which the subject of puerperal fever was fully discussed, several valuable papers being read.

The President appointed as delegates to the State Medical

Society, to meet May 20, at Lincoln, Drs. H. B. Brown, W. S. Watson, J. A. Walker A. M. Bird, C. B. Maclay, S. T. Hurst and W. P. Walker; and as alternates, Drs. J. P. Walker, O. P. Crane, J. W. Spear, A. I. Maclay, I. N. Ellsberry, C. E. Elliot and G. H. Sanford. And as delegates to the American Medical Association, to meet at Atlanta, Ga., May 6, Drs. M. Hurst, L. L. Leeds and J. D. Whitley.

Adjourned to meet in Petersburg, July 24, 1879.

J. A. WALKER, *Secretary.*

